# Level of Physical Activity, Sleep Quality and Use of Personal Protective Equipment of Students at Wroclaw Medical University during the COVID-19 Pandemic

**DOI:** 10.3390/ijerph20032406

**Published:** 2023-01-29

**Authors:** Aureliusz Andrzej Kosendiak, Bartosz Adamczak, Sylwiusz Kontek, Zofia Kuźnik, Michał Roman, Michał Gostkowski, Arkadiusz Niedziółka

**Affiliations:** 1 Department of Physical Education and Sport, Wroclaw Medical University, 51-601 Wroclaw, Poland; 2 Student Scientific Association, Department of Physical Education and Sport, Wroclaw Medical University, 51-601 Wroclaw, Poland; 3 Warsaw University of Life Sciences, Institute of Economics and Finance, Nowoursynowska 166, 02-787 Warsaw, Poland; 4 Department of Econometrics and Statistics, Warsaw University of Life Sciences, Institute of Economics and Finance, Nowoursynowska 166, 02-787 Warsaw, Poland; 5 Faculty of Agriculture and Economics, University of Agriculture in Krakow, 31-120 Krakow, Poland

**Keywords:** COVID-19, medical students, physical activity, sleep, mental health, personal protective equipment

## Abstract

The study was conducted in October 2020 and March 2021 among Wroclaw Medical University students of different years and faculties. The objective of the study was to establish the relationship between some determinants, such as gender and the levels of physical activity, and the quality of sleep of medical students during the pandemic. Ultimately, 696 responses from October and 652 from March were included. To determine the level of physical activity, the International Physical Activity Questionnaire (IPAQ) was used, and for sleep quality, the Pittsburgh Sleep Quality Index (PSQI) was used. The findings pointed to a higher Total MET m/w (metabolic equivalent of task in minutes a week) in men compared to women in both study periods (2020: 1845.8 to 1542.5, *p* = 0.009; 2021: 2040.7 to 1826.6, *p* = 0.025). This was mainly due to a higher Vigorous Exercises MET m/w in men (2020: 837.3 to 635.8, *p* = 0.008; 2021: 773.3 to 490.3, *p* = 0.0006). Moreover, women had a lower quality of sleep resulting from problems in Habitual Sleep Efficiency, Sleep Disturbances, and Daytime Dysfunctions. An adequate level of physical activity and a good night’s rest are the fundaments of health; therefore, it is necessary to determine the causes of their deficiencies in order that we can counteract them.

## 1. Introduction

In December 2019, a novel infectious disease outbreak has occurred in the Chinese city of Wuhan. The virus causing COVID-19 (coronavirus disease) was eventually called the SARS-CoV-2 virus, and it has been rapidly spreading across the world ever since [1,2]. In March 2020, the World Health Organization declared a state of pandemic [3], which led to the implementation of countrywide lockdown measures by the Polish government authorities. An epidemic emergency state in Poland enforced social isolation by banning public or state gatherings of more than 50 people, shutting down cultural events venues, restaurants, bars, and shopping centers, restricting access to churches, gyms, and what is more, educational facilities and other workplaces [4]. It caused a switch to online learning in schools and universities as well as remote work in many industries. The restriction of direct interpersonal interactions was connected with a sudden and rapid need of accommodating to a new, extraordinary reality [5]. Numerous studies show that all of the factors mentioned above could have had a great negative impact on people’s mental health causing increased levels of fear, grief, numbness, anxiety-induced insomnia [6] and an elevated sense of depression or troubles with attention and concentration [7].

The SARS-CoV-2 virus can be transmitted with small liquid particles that are spread with coughing, sneezing, speaking, or breathing by an infected person [8]. Because of the contagious nature of COVID-19, in addition to lockdown measures, people were advised to implement various personal protective equipment (PPE) procedures, such as wearing well-fitted face masks and gloves, using hand sanitizers, and frequently washing hands [9]. A study conducted in Poland shows that most Polish students, in fact, followed these main countermeasures, although their protocol was rarely entirely proper, as for example, many students used disposable masks more than once [10].

All the social, environmental, and economic alterations mentioned above posed another great challenge to people: the maintenance of their physical activity. It became less attainable to the general public as a result of the house confinement in inadequate conditions or locking down sports centers and gyms. Disadvising any unnecessary outdoor activities at the beginning stages of the pandemic, in order to encourage social distancing, was also enforced by shutting down outdoor sports fields [11]. These issues, combined with a commonly weakened motivation, due to a higher level of stress and unexpected lifestyle changes [11,12,13], were the reason of switching toward a more sedentary lifestyle, and therefore, a decreased frequency of exercise [14]. Recent research shows that during the lockdown, medicine students’ total physical activity and metabolic expenditure was reduced, while their sedentary behavior was increased [15]. Another study, conducted amongst nursing students, showed that almost half of the research group gained weight and did not exercise regularly during that period of time [16]. This tendency is even more worrying, as physical activity is well known to be beneficial to general health as it reduces the level of stress, anxiety, and depression [17,18,19]. Therefore, many students developed harmful coping mechanisms, in many cases leading to increased addictive behaviors and even further decreased frequency of physical activity [17,19,20].

In addition to these changes, many studies suggest a negative impact of the COVID-19 pandemic on sleep patterns as well [21,22]. A healthy sleeping routine is a crucial element of maintaining general well-being as it is known to affect the immune system and immunological response. Therefore, the pandemic can cause sleep disturbances and harm the health system’s function [23]. Sleep loss, short sleep duration and other sleep disturbances are associated with a rise of inflammation, as they impair adaptive immunity. It can also reduce a response to vaccines and cause an increased susceptibility to infectious diseases [24], which created a significant threat during the SARS-CoV-2 virus spread. Short-term consequences of sleep disruption can include somatic pain, emotional distress, mood disorders and memory deficits, whereas some of the long-term repercussions include hypertension, cardiovascular disease and weight-related disorders [25]. Moreover, in prepandemic times, multiple researchers examined the sleep hygiene of medical students in particular. They reported an increased level of sleep disturbances and a lower quality of their sleep compared to different control groups [26,27]. It is more concerning because sleep disorders can be correlated with a burnout amongst medical students [28], and the pandemic reality could have worsened the students’ sleep patterns even more.

The aim of the following study is to determine the correlation as well as to characterize the level of physical activity, sleep quality and the use of personal protective equipment by medical students during the COVID-19 Pandemic. The results of the longitudinal study presented later may show various coping strategies with an elevated sense of anxiety and stress that students experienced at this period of time. Examining such factors is the most valid issue in this specific research group, as its future professions in the medical fields will require high resilience to stressors and to the negative effects of isolation during long-hour hospital shifts [29,30,31]. Furthermore, they will be expected to encourage their patients to a healthier lifestyle, also by illustrating it with their own habits, as they perform an essential social function [32,33,34]. Hence, it is substantial to monitor different habits and coping mechanisms of medical-related degree students.

## 2. Materials and Methods

### 2.1. Study Design and Participants

**Figure 1:**
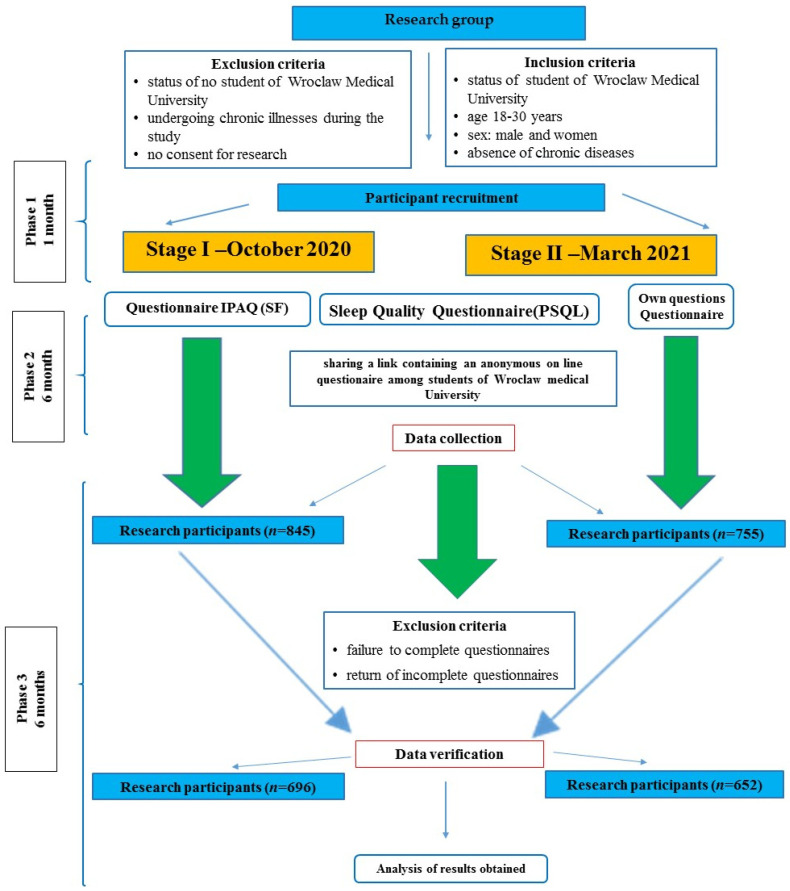
Selection process of the study.

The study was conducted between October 2020 and March 2021. The participant selection process is crucial for a reliably conducted study. The study size was planned to include all eligible students at Wroclaw Medical University having Physical Education classes in the current year in order to collect the most significant data for this cross-sectional study. The exclusion and inclusion criteria, along with the entire process of preparation and selection of participants for the study, are presented in Figure 1.

#### 2.1.1. October 2020

In Phase 1, which took place in October 2020 and lasted for 1 month, students selected as eligible for the study were notified of the invitation via university email and in physical education classes, and the links to the online questionnaire were sent to them. The questionnaire consisted of the International Physical Activity Questionnaire (IPAQ), the Pittsburgh Sleep Quality Index (PSQI), and the authors’ questionnaire, which included metric questions and questions about the COVID-19 pandemic. In Phase 2, 845 responses were collected. In Phase 3, all responses were verified. Responses in which participants failed to complete questionnaires, returned incomplete questionnaires, or where non-compliance in verification questions was identified, were removed. As a result, 696 respondents were accepted after the entire verification process. Possible non-response to all questions in the questionnaire, and the respondents’ chronic diseases were the exclusion criteria, as the results would have been insignificant for this study.

#### 2.1.2. March 2021

In Phase 1, which took place in March 2021 and lasted for 1 month, students selected as eligible for the study were notified of the invitation via university email and in physical education classes, and the links to the online questionnaire were sent to them. The questionnaire consisted of the International Physical Activity Questionnaire, the Pittsburgh Sleep Quality Index, and the authors’ questionnaire, which included metric questions and questions about the COVID-19 pandemic. In Phase 2, 755 responses were collected. In Phase 3, all responses were verified. Responses in which participants failed to complete questionnaires, returned incomplete questionnaires, or where non-compliance in verification questions was identified, were removed. As a result, 652 respondents were accepted after the entire verification process. Possible non-response to all questions in the questionnaire and chronic diseases in the respondents were exclusion criteria, as the results would have been insignificant for this study.

#### 2.1.3. Overall Information

The difference in the number of responses obtained between the genders was expected, as roughly 70% of Wroclaw Medical University students are females.

Wroclaw Medical University trains in all medical professions licensed in Poland. It has more than 6000 students.

Participation in the research was voluntary, anonymous, and had the approval of the Bioethics Committee at the Wroclaw Medical University (No. KB-251/2020).

The diagram below shows the entire process of preparation and selection of study participants (Figure 1).

### 2.2. Level of Physical Activity

The Short Polish version of the International Physical Activity Questionnaire (IPAQ) can be used to measure and compare the physical activity of different populations. We used the short form to assess the level of physical activity among students. As the study shows, the completion of the questionnaire by respondents may exaggerate the results; therefore, all participants of the survey were trained by our research team [35]. The respondents answered the same questions in October 2020 and March 2021. Then, we compared the results with each other. The results of the study were presented in energy expenditure expressed in MET units, i.e., the equivalent of resting metabolism. This is equal to the consumption of 3.5 mL of oxygen per kilogram of body weight per minute. In resting metabolism, MET level = 1. The questionnaire divided the respondents according to the following values: for walking MET = 3.3; for moderate intensity MET = 4; for vigorous activities MET = 8 [36]. Most of the data, unless noted otherwise, were given in minutes a week (m/w).

### 2.3. Sleep Quality

The Pittsburgh Sleep Quality Index (PSQI) [37] is a questionnaire consisting of 19 self-rated questions, concerning the last 4 weeks, that examine seven components of sleep quality, and 5 questions rated by a person sleeping with the respondent (which were excluded in our analysis due to the fact they are not used in the scoring and the fact that most students sleep alone). The score of each component ranges from 0 (lack of an issue) to 3 (the greatest issue). The total score ranges from 0 (the best possible sleep quality) to 21 (the worst possible sleep quality). The assumed value for “good sleep” was less than 5 points in the Total Score. The components are:

⁃ Sleep Duration—0 points for sleeping 7+ hours;
⁃ Sleep Disturbances—score depending on whether there were things that disturbed the continuity of night sleep, e.g., feeling too hot or too cold, bad dreams, uncomfortable breathing;
⁃ Sleep Latency—0 points for latency less than 15 min and 3 for more than 60 min;
⁃ Daytime Dysfunctions—score depending on the number of situations in which lack of nighttime rest affected daytime behavioral disturbances, e.g., eating meals, attending meetings;
⁃ Habitual Sleep Efficiency—score depending on the ratio of actual hours of sleep to time spent in bed;
⁃ Subjective Sleep Quality—score depending on the subjective sleep quality of the respondent;
⁃ Use of Sleep Medication—score depending on how often the respondent took sleep medication.

### 2.4. Own Questions Questionnaires

The questions in the “metrics” section included basic information about the participants: age, gender, body height, body weight, and place of residence according to city size. In addition, the body mass index was calculated. The last part of our questions concerned the COVID-19 pandemic. BMI was calculated using the data declared by individual participants. It was not measured using the bioimpedance method due to the limitations of COVID. This group of questions examined the impact of the pandemic on students in March 2021 after one year of the COVID-19 pandemic. Respondents answered questions about their status, i.e., the restrictions imposed on them, their well-being, and the real impact on themselves. The reliability of the metrics and COVID-19 of the related questions was calculated using Cronbach’s Alpha test.

### 2.5. Statistical Analysis

Excel (Microsoft, Redmond, WA, USA) was used for statistical processing and the search for significant dependencies. The values obtained in the survey had a distribution that differed from the norm (an assessment of the correspondence of the obtained values to the normal distribution of the variation series using the Shapiro–Wilk W-test). A descriptive analysis was performed with frequency and percentages for the qualitative variables and with mean + standard deviation (±SD) for quantitative variables. To compare scores between two variables, the Mann–Whitney U test was used; for three or more variables, the Kruskal–Wallis test was used. For comparison between nominal variables (such as BMI and good/bad sleep quality), the Chi-square (χ^2^) test was performed. The significance level for all the analyses was set at *p* < 0.05.

## 3. Results

Table 1 presents basic information about survey participants. The study was conducted twice on different groups of medical university students. In October 2020, 696 students took part in the survey, and their average age was 20.2 years, while in March 2021, there were 652 students and their average age was 20.4 years. The number of women in the study was initially 527 (75.7%), but in March 2021, this value was 522 (80.1%). The rest of the survey participants were men (in 2020, it was 169, 24.3%; in 2021, it was 130, 19.9%). When determining the place of residence, in both periods of the survey, the largest number of answers was obtained for the city with more than 500,000 inhabitants (in 2020, 206 people, and in 2021, 199 people) and for the rural area (in 2020, 186, and in 2021, 191 people). The rest of the respondents chose the other options from the survey in similar proportions; a city with less than 20,000 inhabitants received 72 responses in 2020 and 63 responses in 2021; a city of 20,000–50,000 received 80 responses in 2020 and 73 in 2021; a similar number of responses was given to the city of 50,000–100,000 option in 2020 and 2021 (73 and 72 people, respectively). For the city of 100,000–500,000, 79 responses were obtained in 2020 and 54 responses were obtained in 2021.

BMI was also measured in the study groups. The average for 2020 was 21.5, while in 2021, it increased to 21.9. The highest percentage in both years was normal BMI (18.5–24.9)—in 2020, it was 543 people (78%), and in 2021, it was 487 people (75%). There were 72 overweight people (BMI > 25) in 2020 (10%), and in 2021, there were 97 overweight people (15%). BMI indicating underweight (<18.5) was shown in 81 people in 2020 (12%), and in 2021, it was shown in 68 people (10%). Members of the Medical University of Wroclaw were asked about their field of study. The largest group was medical students both in 2020 (329 people; 47.3%) and in 2021 (184 people; 28.8%). The second largest group in 2020 was composed of pharmacy (128 people; 18.4%): in 2021, there were 112 people (17.2%). The second largest group of students in 2021 was nursing (128, 19.6%), while in 2020, there were 22 people (3.2%). In 2020 (5.9%), there were 41 people from the medical laboratory field, and there were 29 in 2021 (4.4%). The answers of students from dietetics in 2020 and 2021 were similar (31 people, 4.5% and 33 people, 5.1%, respectively). From physiotherapy, 78 people (11.2%) responded to the survey in 2020, and 69 people (10.6%) responded in 2021. There were 10 students of dentistry in the survey in 2020 (1.4%), and in 2021, there were already 27 people (4.1%). In 2020, people from the fields of midwifery (17, 2.4%), paramedic (28, 4%), and public health (12, 1.7%) also responded to the survey. In 2021, there were 25 people (3.8%), 24 people (3.7%), and 21 people (3.2%), respectively, from these majors. We checked how the respondents assess the impact of the pandemic on well-being in both time periods. In both cases, most people said that the pandemic had no impact (in 2020, 242 people, 34.8%; in 2021, 266 people, 40.8%) and that it was hard to assess the impact of the pandemic on their well-being (in 2020, there were 385 such answers, 55.3%, and in 2021, there were 320, 49.1%). The smallest group was composed of people for whom the pandemic had a positive impact on well-being both in 2020 and 2021 (18 people, 2.6% and 19 people, 2.9%, respectively). In 2020, 51 people (7.3%) responded that the pandemic had a negative impact on their well-being, and in 2021, this number had decreased to 47 people (7.2%). During the study, mental condition during the COVID-19 pandemic was examined. The majority of people felt overwhelmed in 2020 (354, 50.4%) and in 2021 (242, 37.1%). A large group of people also felt the same (in 2020 106 people, 15.2% and in 2021 150 people, 23%). The other answers to this question were: ‘I feel lonely’ (in 2020 53 responses, 7.6% and in 2021 60 responses, 9.2%), ‘I feel anxious’ (in 2020, 85 responses, 12.2% and in 2021 32 responses, 9.2%), ‘I am more stressed’ (in 2020 none, 0% and in 79 people, 12.1%), ‘I cannot function’ (52 people, 7.5% in 2020 and in 2021 42 people, 6.4%) and ‘I feel happy’ (in 2020, 49 people, 7% and in 2021, 47 people, 7.2%).

Table 2 describes the amount of different personal protective equipment (PPE) among medical students in October 2020 and March 2021. Each year, most of the survey participants were washing their hands (in 2020 682, 98% and in 2021 644, 98.8%) and were using face masks outside the home (in 2020 690, 99.1% and in 2021 637, 97.7%). A high number of students also declared that they use hand sanitizers in 2020—629 (90.4%) and in 2021—571 (87.6). Overall, 123 people (17.7%) in 2020 were using gloves outside the home and in 2021, this number was 86 (13%). None of the study participants admitted that they did not use any form of PPE. The rest of the answers reached a score under 5% of study participants.

Table 3 shows the score in MET minutes a week (m/w) for different activities in 2020 and 2021. It was measured how much people walked—in 2020, the average MET m/w was 840.8 (median 627), while in 2021, this value increased to 1181.3 (median 792); this difference is statistically significant (*p* < 0.0001). Another statistically significant result (*p* = 0.0051) was the increase in total physical activity in March 2021 (average MET m/w was 1865.9, median 1386) compared to October 2020 (average MET m/w was 1615.7, median 1332). People practicing vigorous physical activity scored lower MET m/w in 2021 (546.7, median 160) than in 2020 (635.8, median 240). This result is significant (*p* = 0.023). For moderate physical activities, people scored on average MET m/w 139 in 2020 and 138 in 2021, but the difference between them was statistically insignificant (*p* = 0.053). Qualitative physical activity level among medical students was also examined. In October 2020, 541 students (77.7%) were ‘inactive’, 74 of the survey participants were minimally active (10.6%), and only 81 (11.6) of them reached health-enhancing physical activity (HEPA). In 2021, the results were slightly different. Overall, 526 (80.7%) students were inactive, 63 of them (9.3%) were minimally active, and the same amount of people achieved HEPA.

Table 4 presents the dependences of gender on the achieved MET m/w scores for various activities in two different periods during the pandemic. MET m/w for walking among both females and males had grown from October 2020 to March 2021. Females were scoring 835.0 on average in 2020 and 1207.1 in 2021. The result is statistically significant (*p* < 0.0001). Males achieved higher scores than women in both years (for 2020—819.2 and for 2021 1077.3), but these differences are statistically insignificant (in 2020 *p* = 0.22 and in 2021 *p* = 0.25). The increase in male walking MET m/w during the pandemic is statistically significant (*p* = 0.019). In moderate physical activities, women reached on average MET m/w 135.8 in 2020 and 129.2 in 2021. The decrease of that value is statistically significant (*p* = 0.023). Males reached on average a higher score in both years (in 2020 149.6, and in 2021 173.3), but that difference was statistically significant only in 2021 (*p* = 0.038 for 2021 and in 2020—*p* = 0.47). The increase in the values achieved by men was insignificant (*p* = 0.33). Male and female MET m/w for vigorous physical activity decreased during the pandemic. Women scored in October 2020 635.8 and in 2021 490.3 (*p* = 0.025), while men in 2020 scored 837.3 on average and 773.3 in 2021 (*p* = 0.039). It is worth noting that the median MET m/w for vigorous physical activity for women in 2021 was equal to 0. The difference between both genders in 2020 and 2021 was also statistically significant (in 2020, *p* = 0.008 and in 2021, *p* = 0.0006). Adding everything up, the total MET m/w for females was 1542.5 on average in 2020 and 1826.6 on average in 2021. An increase in that value was statistically significant (*p* = 0.0077). Men reached on average in both years higher scores than women; the difference was statistically significant *p* = 0.009 and the same in 2021 with *p* = 0.025. Men had 1845.8 on average in 2020 and 2040.7 in 2021, but that increase is not statistically significant (*p* = 0.15).

Table 5 presents the data associated with sleep quality scores reached in the group of survey participants in 2020 and 2021. Sleep duration was leveled on a value of 0.37 in 2020. In 2021, this value was already 0.44, so the quality of sleep deteriorated during the pandemic, which is statistically significant (*p* = 0.022). Sleep latency and sleep disturbances also became worse, in 2020, scores reached by survey participants were 1.41 for sleep latency and 1.16 for sleep disturbances, and in 2021, they were 1.46 for sleep latency and 1.21 for sleep disturbances, but those data were not statistically significant (consecutively, *p* = 0.20 and *p* = 0.054). Daytime dysfunctions lowered from 1.53 in 2020 to 1.45 in 2020, but that score is not significant. From 2020 to 2021, habitual sleep efficiency slightly raised from 0.34 on average to 0.36, but that increase was not statistically significant. Similarly, subjective sleep quality was higher in 2021 with a score 0.95 on average than in 2020 (0.92 on average), but that dependence was insignificant. The use of sleep medication reached a similar score in both periods (in 2020 0.17 and in 2021 0.16), but those data were also insignificant. Overall, 321 medical students (46%) answered that their sleep quality is good, and 375 (54%) survey participants claimed that their sleep quality is poor. That was maintained in 2021 where 314 students (48%) rated their sleep as good, and 338 students (52%) rated it as poor.

Table 6 presents the dependence of various aspects of sleep quality among women and men in 2020 and 2021. Sleep duration for both men and women became worse (women in 2020—0.35 and in 2021—0.43; men in 2020—0.42 and in 2021—0.48), but only for women was that change significant *p* = 0.029 (for males *p* = 0.22). The differences between gender during both periods of time were statistically insignificant (in 2020, *p* = 0.25 and in 2021, *p* = 0.37). Sleep disturbances among males were constant between 2020 and 2021 with an average of 1.09, and the change was not significant (*p* = 0.43). For females, there was an increase in this value during the pandemic from 1.18 in 2020 to 1.25 in 2021, which is statistically significant. In both 2020 and 2021, females had more sleep disturbances than males, which is significant (in 2020 *p* = 0.04 and in 2021 *p* = 0.0046). The value of sleep latency also increased during the pandemic for both males and females: men’s were higher more in both periods than women, but none of those differences were significant. In 2020, men had a lower score for daytime dysfunctions (1.30) than women (1.60), which is statistically significant (*p* = 0.0009), and in 2021 (men 1.12 and women 1.53) it was also significant (*p* < 0.0001). The decrease in that value for both genders during the pandemic is statistically insignificant (for women, *p* = 0.13 and for men, *p* = 0.07). Female habitual sleep efficiency was almost constant during the pandemic (in 2020, 0.37 and in 2021, 0.36), which is insignificant (*p* = 0.28). Men had identical values to women in 2021 (0.36), but in 2020, they scored 0.25. An increase in this value was insignificant. In 2021, values for men and women were not significant (*p* = 0.49), but in 2020, women had a significantly (*p* = 0.0069) higher score in habitual sleep efficiency than men. For men, subjective sleep quality was constant during the pandemic (0.96), but that was insignificant (*p* = 0.43). For women, that amount has increased from 0.90 in 2020 to 0.94 in 2021. This difference was statistically insignificant (*p* = 0.21). There was no difference between men’s and women’s subjective sleep quality in 2020 and in 2021 (consecutively, 0.33 and 0.47). The amount of use of sleep medications taken by women was constant during the pandemic (0.17), which was not statistically significant (*p* = 0.20), and there was a decrease in this number for men (in 2020, 0.16 and in 2021, 0.13), but that was also insignificant. There was no statistical difference between the use of sleep medication and gender in 2020 (*p* = 0.50) and in 2021 (*p* = 0.38).

In 2021, there was a significant difference between sleep disturbances and daytime dysfunctions for women—0.27 (*p* > 0.0001) and for men—0.24 (*p* = 0.005). In 2020, this difference was also significant for women—0.21 (*p* > 0.0001) but not for men—0.035 (*p* = 0.32).

Table 7 shows the differences between PSQI components and the impact of the pandemic on well-being in October 2020 and March 2021. Sleep duration for people who described the impact as ‘negative’ was the shortest in both periods (0.20 for October and 0.36 for March). The highest score in 2020 was for students with the positive impact of the pandemic on their well-being with a value of 0.47, but in 2021, it decreased to 0.37. In 2021, the highest value was for survey participants who answered ‘hard to decide’—0.45 (in 2020, it was 0.40). People who felt no impact on their well-being scored in 2020 on average 0.35 and in 2021—0.44. None of the above results are statistically significant (in 2020, *p* = 0.18 and in 2021, *p* = 0.85). In 2020, medical students had the lowest sleep disturbances for the ones who felt the negative impact of the pandemic (0.96). In 2021, that value increased to 1.13. For other groups in 2020, the results were the following: positive impact on well-being: 1.00; no impact on well-being: 1.12; hard to decide: 1.22. In 2021, results were slightly different: positive impact on well-being: 1.32; negative impact on well-being: 1.13; hard to decide: 1.33. The results were significant both in 2020 (*p* = 0.048) and in 2021 (*p* = 0.0026). Sleep latency scores in 2020 and 2021 were very diverse. For people who declared a positive impact, that value was 1.76 in 2020 and 1.26 in 2021; for students who felt a negative impact, the results were 1.12 in 2020 and 1.49 in 2021; ‘no’ impact was felt in 1.39 in 2020 and 1.41 in 2021; the values for option ‘hard to decide’ were for 2020—1.45 and for 2021—1.51. For both years, the results were statistically insignificant (consecutively, *p* = 0.056 and *p* = 0.46). Differences between daytime dysfunctions and the impact of the pandemic on well-being were clearly marked and significant (for both 2020 and 2021, *p* < 0.0001). Among students who could not decide, the results were in 2020—1.64 and in 2021—1.63. Survey participants who declared no impact on their well-being scored 1.49 in 2020 and 1.32 in 2021; for the positive impact, the value was 1.12 in 2020 and 1.47 in 2021, and for the negative impact, it was 0.98 in 2020 and 0.94 in 2021. Habitual sleep efficiency had differed within the impact of the pandemic on well-being in the following way: positive impact in 2020—0.12 and in 2021—0.47; negative impact in 2020—0.25 and in 2021—0.34; no impact on well-being in 2020—0.31 and in 2021—0.33; hard to decide in 2020 and 2021—0.38. Results were for both years statistically insignificant (consecutively, *p* = 0.17 and *p* = 0.79). In 2020, the difference between subjective sleep quality and the impact of the pandemic on well-being was significant (*p* = 0.0039) and the results follow: positive impact—0.71; negative impact—0.65; no impact—0.95; hard to decide—0.94. In 2021, this difference was statistically insignificant (*p* = 0.14)—for positive impact: 0.79; for a negative impact: 0.83; for no impact: 0.91 and for hard to decide: 1.0. In 2021, there was a significant difference between the impact of the pandemic on the well-being and using sleep medications (*p* = 0.03). The highest score was for students who felt a positive impact on their well-being (0.32) and who declared that it is hard to decide (0.22). The lowest values were found for students who answered that the impact is negative (0.11) or there is no impact (0.10). In 2020, that difference was insignificant (*p* = 0.45). In the total score of sleep quality, the lowest value on average was for a group of survey participants with the answer ‘negative’ impact: in 2020—4.27 and in 2021—5.19. The highest score was reached for the ‘hard to decide’ option (in 2020—6.20 and in 2021—6.50). For positive impact, the results were 5.59 in 2020 and 6.00 in 2021; for no impact on well-being: in 2020—5.78 and in 2021—5.62.

Chi-square tests comparing the sleep quality (good or bad sleep) with the impact of the pandemic on well-being were also carried out. In October 2020 (χ^2^ = 16.0, *p* = 0.0011), almost the same amount of people who described the impact as positive slept well (1.2%) and poor (1.3%). More people slept well (5.2%) rather than poor (2.2%) in a group of people who declared that the impact was negative. For the groups who could not define the impact or felt there was no impact, more people had assigned their sleep as good (consecutively 16.8%; 23.0%) rather than bad (consecutively 18.0% and 32.4%). In March 2021 (χ^2^ = 10.9, *p* = 0.01) for all statements, in general, of the impact of the pandemic on well-being, there was a higher percentage of people who slept well rather than who did not: positive impact—good sleep: 1.8% and poor sleep: 1.1%; negative impact—good sleep: 5.1% and poor sleep: 2.0%; none impact—good sleep: 25.1% and poor sleep: 15.7%; hard to decide—good sleep: 25.2% and poor sleep 24.0%.

Table 8 presents the differences between mental conditions and sleep quality score components during two different periods of the pandemic. Sleep duration did not differ significantly depending on the mental condition (*p* = 0.28 in 2020 and *p* = 0.13 in 2021). People who felt the same reached 0.25 in 2020 and 0.39 in 2021. For the answer ‘I feel lonely’, the results were 0.49 in 2020 and 0.52 in 2021. People who felt anxious in 2020 reached 0.40 for sleep duration and 0.25 in 2021. The highest score was for the answer ‘I am stressed’ in 2021—0.62. People who claimed that they cannot function reached 0.42 in 2020 and 0.55 in 2021. For answers ‘I am overwhelmed’ and ‘I am happy’, the results in 2020 were the same—0.37, and in 2021, they were slightly different (consecutively 0.40 and 0.43). People who felt the same had the lowest sleep disturbances score (in 2020—1.05 and in 2021—1.02). The other results for this component follow: I feel lonely: 1.17 in 2020 and 1.40 in 2021; I feel anxious: 1.28 in 2020 and 1.31 in 2021; I feel more stressed: 1.28 in 2021; I cannot function: 1.08 in 2020 and 1.52 in 2021 (highest value in 2021); I am overwhelmed: 1.18 in 2020 and 1.24 in 2021; I feel happy: 1.16 in 2020 and 1.13 in 2021. The results were statistically significant for 2021 (*p* = 0.00076) but not for 2020 (*p* = 0.17). Sleep latency differed significantly depending on mental condition in 2020 (*p* = 0.00073): I feel the same: 1.33; I feel lonely: 1.92; I feel anxious 1.52; I cannot function: 1.31; I am overwhelmed: 1.32; I feel happy: 1.61. In 2021, results were statistically insignificant (*p* = 0.29): I feel the same: 1.30; I feel lonely: 1.58; I feel anxious: 1.66; I feel more stressed: 1.47; I cannot function: 1.71; I am overwhelmed: 1.46; I feel happy: 1.45. There was a strong statistically significant difference between daytime dysfunctions and mental conditions in both years (in 2020, *p* = 0.00011 and in 2021, *p* < 0.0001). The lowest score was for the answer ‘I feel the same’ in 2020—1.10 and in 2021—1.04. The highest score in 2020 was set for ‘I feel happy’—1.78 (in 2021 dropped to 1.47), and in 2021, the highest value was for ‘I cannot function’—1.86 (in 2020—1.67). For the ‘I am overwhelmed’ statement, in 2020, the score was 1.60 and in 2021, it was 1.57. ‘I feel lonely’ had a higher score in 2020 (1.62) and in 2021 (1.55) than ‘I feel anxious’ (consecutively 1.47 and 1.22). Subjective sleep quality in 2020 compared to mental condition reached values in the range of 0.84–0.98 (the lowest for the ‘I feel the same’ and ‘I feel happy’ and the highest for the ‘I feel lonely’). Those results were insignificant (*p* = 0.51). In 2021, the same comparison was statistically significant (*p* = 0.00028). The highest score reached students who answered ‘I feel more stressed’—1.22. The lowest values were for the ‘I feel the same’—0.79 and for the ‘I feel anxious’—0.78. Other results follow: ‘I feel lonely’: 0.98; ‘I cannot function’: 1.14; ‘I am overwhelmed’: 0.92 and ‘I feel happy’: 1.06. The study showed a significant difference between mental condition and using sleep medications in 2020 (*p* = 0.029). The highest score was reached by people who answered, ‘I feel happy’ (0.39). ‘I feel anxious’ reached 0.25, ‘I feel lonely’ reached 0.19, ‘I feel the same’ reached 0.16, ‘I am overwhelmed’ reached 0.13, and ‘I cannot function’ reached 0.04 (the lowest score). In 2021, the results were insignificant (*p* = 0.99), and the values were in the range of 0.13 to 0.19. In Total Score, the data collected in both periods of time were statistically significant (in 2020, *p* = 0.00097; in 2021, *p* < 0.0001). The ‘I feel the same’ option had the lowest score in both years: in 2020—5.03 and in 2021—4.37. In 2020, the highest value on average was reached by people answering, ‘I feel lonely’ (6.83), but in 2021, they reached only 5.73. In 2021, the highest average score was for the ‘I cannot function’ answer (6.64); in 2020, that option had a score of 5.77 on average. ‘I feel anxious’ reached 6.14 in 2020 and 4.84 in 2021. ‘I am more stressed’ was also examined in 2021 and reached 6.04 on average in the total score. For ‘I am overwhelmed’ and ‘I feel happy’, the results were consecutively: in 2020—5.91, 6.41; in 2021—5.48, 5.55.

Chi-square tests comparing the sleep quality (good or bad sleep) with the impact of the pandemic on well-being were also carried out. In October 2020 (χ^2^ = 18.9, *p* = 0.0021), the same number of contestants (3.7% of the survey for each year) from the group ‘I cannot function’ had assigned their sleep as good and bad. The biggest group of the survey participants answered that they are overwhelmed. Overall, 23.1% of participants had good sleep, but more of them slept poorly (27.3%); 7.0% of the study group felt happy but only 2.7% of them classified their sleep as good (4.3% had poor sleep). More answers for the poor sleep than the good sleep were found in the ‘I feel anxious’ group (7.2% slept poorly and 5.0% well) and in the ‘I feel lonely’ group (2.2% slept well and 5.5% poorly). People for whom mental condition did not change and answered ‘I feel the same’ slept more often well (9.3%) than poorly (5.9%). In March 2021 (χ^2^ = 25.5, *p* = 0.00027), among groups of people who answered ‘I feel the same’ and ‘I feel happy’, there was a higher percentage of people who slept well rather than those who slept poorly (for ‘I feel the same’, 16% slept well and 7.1% slept badly, and for ‘I feel happy’, 4.4% slept well and 2.8% slept poorly). Similarities can be seen in groups of survey participants who felt anxious (3.2% had slept well and 1.7 had slept poorly); were lonely (4.8% had good sleep quality and 4.4% did not); and were overwhelmed (21.5% slept well and 15.6% did not). People who declared that they cannot function more often slept poorly (2.3% slept well and 4.1% slept poorly); also, people who felt more stressed more often had their sleep assigned as poor (6.9%) rather than bad (5.2%).

Table 9 presents the linking of the area of residence in terms of the number of inhabitants with the sleep quality score sub-units. There are no statistically significant differences between residence area and sleep duration in both periods of the survey (for 2020, *p* = 0.58 and for 2021, *p* = 0.31). Scores reached in 2020 are 0.26 for cities <20,000, 0.34 for cities 20,000–50,000, 0.35 for cities 50,000–100,000, 0.36 for cities 500,000+, 0.40 for rural areas, and 0.47 for cities 100,000–500,000. In March 2021, values were 0.24 for cities <20,000, 0.41 for cities 100,000–500,000, 0.42 for cities 500,000+, 0.46 for cities 50,000–100,000, 0.47 for cities 20,000–50,000, and 0.51 for the rural areas. Sleep disturbances also did not differ between different urban areas (for 2020 *p* = 0.27 and for 2021 *p* = 0.055). In 2020, the highest sleep disturbances were in cities 50,000–100,000 (1.32) and in cities 20,000–50,000 (1.23), and the lowest value was for the rural areas (1.08). In 2021 cities 20,000–50,000 was still the highest-ranked place in that domain (1.35), and cities 500,000+ had the second-highest score (1.33). The lowest value was in cities 100,000–500,000 (1.11). The difference between sleep latency and area of living is not significant for 2020 (*p* = 0.73) and for 2021 (*p* = 0.48). The lowest score was reached in the rural areas for both years (in 2020—1.32 and in 2021—1.35). The highest score was for cities with 20,000–50,000 in 2020 (1.51), but in 2021, it scored only 1.42. The highest score in 2021 was reached by answers made by people living in cities 100,000–500,000 (1.61); in 2020, the value of that option was 1.43. For cities <20,000, the results were 1.46 in 2020 and 1.41 in 2021. For cities 50,000–100,000 and cities 500,000+, the scores were consecutively 1.47 and 1.42 in 2020 and 1.54 and 1.52 in 2021. Daytime dysfunctions differed between the areas of living, and the results were significant in both years (in 2020, *p* = 0.043 and in 2021, *p* = 0.044). In 2020, the biggest daytime dysfunctions were set in cities 500,000+ (1.66) and in cities 50,000–100,000 (1.60). The lowest score was in rural areas (1.34). Cities <20,000 reached a 1.51 score, the value in cities 20,000–50,000 was 1.53, and in cities 100,000–500,000, the score was 1.58. The arrangement of the data has changed for 2021, listing the scores from highest to lowest: cities 50,000–100,000: 1.68; cities 100,000–500,000: 1.56; cities 500,000+: 1.50; cities 20,000–50,000: 1.41; rural areas: 1.39; cities <20,000: 1.13. For habitual sleep efficiency in 2020, depending on the area of residence, scores were within a narrow range of values from 0.28 (reached in cities 100,000–500,000) to 0.38 (reached in cities 20,000–50,000). That results were statistically insignificant (*p* = 0.64). In 2021, the scores were also close to each other. The lowest value was reached in cities 100,000–500,000 (0.28) and the highest score was in cities 50,000–100,000 (0.42). These results were statistically insignificant (*p* = 0.68). Statistically significant results were achieved while comparing living areas with the use of sleep medications (in 2020, *p* = 0.016 and in 2021, *p* = 0.048). In October 2020, the lowest usage of sleep medication was in cities 50,000–100,000 (0.03). The other results follow: rural area: 0.10; cities <20,000: 0.19; cities 20,000–50,000: 0.21; cities 100,000–500,000: 0.32; cities 500,000+: 0.19. In 2021, the areas with the lowest usage of sleep medications were rural areas (0.06) and cities <20,000 (0.08). Higher results were achieved in cities 100,000–500,000 (0.17), and the highest scores were in the cities 20,000–50,000 (0.21), cities 50,000–100,000 (0.22), and cities 500,000+ (0.25). For the total score in rural areas in 2020, the sleep quality value was 5.39 and in 2021, it was 5.71. For cities <20,000, results were 5.85 in 2020 and 5.19 in 2021. Cities 20,000–50,000 and cities 50,000–100,000 reached higher scores in 2020 and 2021 (consecutively, 6.20 and 6.23 in 2020 and 6.03 and 6.71 in 2021). For cities 100,000–500,000 and cities 500,000+, values were lower but not the lowest in both periods of the survey (consecutively 6.01 and 6.09 in 2020 and 6.19 and 6.33 in 2021). In 2020 and 2021, results were insignificant (for 2020 *p* = 0.11 and for 2021 *p* = 0.07).

In 2020, there was a significant difference between daytime dysfunctions and physical activity level (*p* = 0.015). People who were inactive reached a value 1.58 of for daytime dysfunctions, students moderately active reached 1.41 and HEPA scored only 1.27.

In 2021, sleep duration differed depending on the BMI (*p* = 0.0009). Underweight people scored 0.21, the normal BMI result was higher (0.43), and the highest value was for overweight people (0.67).

Furthermore, the study participants were divided into two groups—clinical and non-clinical—depending on their patient contact. Medical laboratory, public health, dietetics, and pharmacy were assigned to the non-clinical group due to low or no contact with patients. Physiotherapy, medicine, dentistry, nursing, paramedic, and midwifery were determined as clinical faculties. A comparison of physical activity levels between clinical and non-clinical faculty students was made for 2020 and 2021 separately. Most of the results were irrelevant. Only in 2020, moderate MET m/w *p*-Value indicated a statistically significant differences (*p* = 0.042). Students from non-clinical faculty scored more (157.2) than clinical faculty students (131.1). This comparison in March 2021 was not significant. Sleep quality was also examined in comparison with the nature of the faculty. In October 2020, daytime dysfunctions were higher for non-clinical students (1.65) than for clinical students (1.47), and that difference was statistically significant (*p* = 0.042). In March 2021, the results were insignificant (*p* = 0.406). A comparison made for other sleep quality components, which were statistically insignificant for both October 2020 and March 2021.

## 4. Discussion

### 4.1. Anthropometric Data, Place of Residence during the Epidemic, and the Impact of Pandemic

This study evaluated the influence of the COVID-19 pandemic related to its restrictions amongst Wroclaw Medical University students on their use of personal protective equipment, physical activity levels, and changes in their quality of sleep. The research group consisted mostly of women, as they constitute the majority of students at this university. In 2020 and 2021, the study respondents most frequently lived in villages and the biggest cities, while a slight increase in population was noticed in rural areas, cities of more than 500,000 inhabitants and of 50,000–100,000 inhabitants. These results are partially consistent with the demographic situation in Poland up to 2021 presented by the Government Population Council [38]. It is stated that the population growth continued in villages and in the largest cities, and the trend of the population decline was observed in the population of medium-sized towns; the latter is not entirely consistent with our study, as the population of 50,000–100,000 inhabitants cities increased. These migrations could have been the result of seeking better access to education and health care but also countrywide lockdowns causing many students to return to their family homes instead of living alone in another city [39].

As for the BMI analysis, a significantly increased number of overweight people was recorded (from 10% in 2020 to 15% in 2021). A decline in underweight BMI was also observed (from 12% to 10%). Therefore, both in the first and the second survey, abnormal BMI level was present in approximately 23.5% of our respondents. Despite the increase in overweight people, it is still a positive result compared to the multiple studies of different populations, e.g., in Poland (36.3%) [40] and Spain (30.3%) [41]. However, the body mass index is not always an accurate tool for measuring excessive body fat, as it does not distinguish fat from fat-free mass such as muscles and bones [42]. Therefore, some students might have been misclassified as overweight when in fact they were athletically built with the correct weight.

Lockdown measures, quarantine, and expanded isolation could have had very negative effects on students’ mental health, causing confusion, anger, and post-traumatic stress symptoms [6,7,43]. The research also examined the general perception of the Pandemic’s impact on the students’ well-being. Interestingly, the majority of people in both study periods stated that it had no impact (on average 37.8%) or it was hard to decide (52.2%), while the minority perceived the impact as positive (2.75%). Additionally, they were asked to access their present mental condition, and most of the students admitted to feeling overwhelmed (50.4% in 2020 and 37.1% in 2021), which would be contradictory to the answers to the previous question. However, the study had not taken place before the pandemic, so it is possible that some of the respondents may have felt in this negative way constantly before the first survey took place. On the other hand, the second most frequently chosen answer to this question, both in 2020 and 2021, was ‘I feel the same’, and it would be consistent with the average perception of the pandemic’s impact on well-being as non-existent or hard to determine. The fact that there is a significant growth of feeling more stressed (from 0 participants in 2020 to 79 in 2021) is also worth mentioning, and multiple studies prove this tendency as well [44,45].

### 4.2. Using Personal Protective Equipment during the Pandemic

During the survey, the participants were asked if they had used any personal protective equipment (PPE). None of the students chose the answer “I do not use any”, which means every respondent did use at least one type of protection on a daily basis. The vast majority reported using face masks outside the home (from 99.1% to 97.7%), which might have been enforced by the government regulations making it mandatory in closed spaces [46]. It is a slightly higher outcome than in other studies, where the result varies around 90% [47], which could be due to the higher likelihood of compliance to lockdown restrictions by medical students, which is elaborated later on in the discussion part. The same amount applied to washing hands, although it may be disturbing that approximately 2% of the participants did not choose this option, as they will be a part of the medical staff in the future, and their patients’ life and health will rely on their personal hygiene as well [48,49]. These results are rather consistent with a Chinese adults study group (96.8%) [50]. However, they are highly more optimistic than in an adult Thai population, where more than 15% of the respondents reported not washing their hands at all [51]. Another commonly used protective measure was using hand sanitizers, which is understandable, as they became easily accessible in public places during the pandemic. Using goggles was rather rare in our study sample (1.25% on average), while in a Portuguese population, up to 12% admitted to using them [47].

### 4.3. Physical Activity during the Pandemic

All of the changes caused by the pandemic affected physical activity as well. It became less attainable to people as a result of shutting down sports facilities, discouraging access to fresh air at the very beginning of the pandemic, and not having appropriate conditions to exercise during home confinement. People were forced out of schools, universities, and offices, concurrently switched to e-learning and online work, so that they had to modify their lifestyles and habits. All these factors led to increased sedentary behavior [52]. Physical education classes were also performed remotely, often asynchronously, leaving many students with the lack of motivation and the access to proper equipment necessary to maintain physical activity. In the result, it led to a decrease in its frequency.

The presented research assessed different types of physical activity amongst the students of medical degrees. Statistically significant results show that the minutes spent on walking, as well as the total ratio of the expended energy (MET), increased from October 2020 to March 2021, which is a positive finding in comparison to other studies showing a declining tendency, where walking time decreased from 40.7% to 24.2% of the total time spent on physical activity [52]. Additionally, men scored a higher total MET m/w in comparison to women in both years. Although the overall moderate and vigorous physical activity, statistically insignificant, decreased in this period of time, we found higher scores of vigorous exercising by men next to women to be statistically significant. Especially the difference in vigorous physical activity between the genders is worth noting. The majority of women were not engaged in such exercises, as indicated by a median of 0 in March 2021 (400 in men). This might explain the difference in Total MET m/w between the genders, which could be due to women’s lower engagement in vigorous physical exercises, which clearly reflects the total level of physical activity. The tendency of decreased vigorous exercising during the pandemic was also reported in an Italian adult population (from 267.15 to 143.17), and it also shows a drastically lower score in their intense physical activity in relation to our study sample (from 635.8 to 546.7) [53]. The mentioned tendency was also observed amongst adults living in the USA, with a lower level of vigorous exercising as well (from 335 to 276.39) [54]. Higher scores in Total MET achieved by men (722.44) than women (1086.5) in the Italian study sample were also consistent with our findings, although the Total MET level was noticeably lower when compared to our study’s results in both groups. However, a study conducted on Spanish students showed an increased level of vigorous and moderate physical activity before and during the lockdown [55]. Previously mentioned tendencies may have been caused by the inadequate access to professional equipment and sports centers that are usually necessary for exercising more intensively, whereas walking was still an attainable way of compensating the elongated seated time, relieving stress and taking care of one’s general health condition [17,18].

The study also presented an increased number of physically inactive students (from 77.7% in 2020 to 80.7% in 2021), which was already worryingly high at the beginning of the study, and an even more concerning low rate of the health-enhancing physical activity (HEPA) that decreased even further: from 11.6% to 9.7% in 2021. It means that on average, only 11% of all the students had exercised enough to have an actual positive impact on their health. The number of physically inactive people in our study was more than fifteen times higher than in the population of middle-aged Brazilians (4.6% during the pandemic) [56], and the HEPA rate was three times lower comparing to the score of Swiss office workers during the pandemic [57]. This is an alarming result and should be taken into consideration during the future education of medical students. The tendency of decreased physical activity during the COVID-19 pandemic is also supported by numerous studies [58,59,60], although there are examples of communities which were not affected by lockdowns in this negative way, such as previously mentioned Swiss office workers [57]. However, the level of physically inactive students presented above is drastically higher than pre-pandemic studies suggest. They state that approximately half of the medical students were physically active (49.5%) [61], whereas our study indicates half of this value. Another pre-pandemic study shows that more than one-half (61%) of U.S. medical students had adhered to health-related physical activity recommendations before the pandemic, which is a tremendously higher rate than the obtained level of HEPA in our study [62]. The insufficient time spent on physical activity may be a result of many barriers not only caused by the pandemic countermeasures but also the characteristics of this research group. The most common of them, before the pandemic, were the lack of time due to excessive need of studying, inconvenient schedules of exercise facilities and tiredness [63].

### 4.4. Sleep Quality during the Pandemic and Dependence on Various Factors

The goal of this research was to determine the pandemic’s impact on students’ sleep quality as well. A healthy sleeping routine is an important element of preserving good health, as its disturbances are associated with increases in inflammation, memory deficits, somatic pain, and in the long term, they can lead to cardiovascular diseases [24,25]. The study participants were asked about their sleep quality in a few categories, within which the higher achieved score indicated the poorer sleep quality was. We can observe a statistically significant increase in unhealthy sleep duration from 2020 to 2021 for both men and women. Sleep disturbances were significantly worse for women. In addition to this, in 2020, women had a worse score in habitual sleep efficiency than men (it is the ratio between one’s actual sleep time and the time spent in bed). A study conducted amongst Brazilian adolescents also presented that during the pandemic, females experienced poorer sleep quality in comparison to males [64]. This tendency suggests that women generally had more issues with their sleep quality than men during the pandemic, which is consistent with the studies conducted before the COVID-19 timeframe as well [65,66].

The overall assessment stated that more students experienced a poor rather than a good sleep quality, and higher scores were maintained in both years on a similar level (54% in 2020 and 52% in 2021). It is consistent with other studies from the pandemic, for example, amongst the Greek medical students, as more than half of the respondents evaluated their sleep as being of poor quality (52.4%) [67], whereas a multinational study of medical students indicated poor sleep quality amongst 73.5% of them [68], which is an even more alarming result. This trend was also present in a large study sample consisting of middle-aged health-care workers (in a total number of 1111 participants), with their average total score of PSQI even higher than the score obtained among students in our study, indicating severe sleep disturbances amongst medical professionals (6.64 compared to 5.9). Additionally, in a study of an elder adults population, the total score of PSQI, during the pandemic, was similar to our results (5.69). Although sleep disturbances within this group were more prominent than amongst the students, daytime dysfunction was more severe within a younger study sample [69]. The former may be due to a naturally higher prevalence of sleep disorders within the elder age group [70]; however, healthy older adults without sleep disorders can expect to be less sleepy during the daytime than young adults [71]. Other findings were not statistically significant, but the noticeable tendency in most categories presented the sleep quality becoming worse during the pandemic. These results align with multiple studies on the matter which suggest that in comparison to the pre-lockdown period, there was a shift to a later bedtime and waking time with a reduction in nighttime sleep and an increase in daytime napping [21]. However, the latter was not observed in our study, as the daytime dysfunctions did not worsen significantly. Sleep disturbances are also believed to be strongly connected with anxiety, depression, and suicidal behavior; hence, it is crucial to monitor these changes, especially during such stressful times as the pandemic [72].

Moreover, our study shows a clear difference between the perceived impact of the pandemic on the sleep quality. Surprisingly, the negative impact of the pandemic was strongly associated with the best sleep quality. It was statistically significant in several categories, such as the lowest sleep disturbances level (although it increased in 2021), the least daytime dysfunctions, and the best subjective and total sleep quality. There is not sufficient data to elaborate on these results at this moment; therefore, further research in this area would be recommended to properly interpret these findings. In the categories mentioned above, the worst effect on sleep quality was observed among people who said that the impact of the pandemic made it hard to decide whether it was positive for them. We believe it can be connected with the psychological denial of the adverse effects of the pandemic amongst these participants, as it is a known defense mechanism during that time [73]. Therefore, another study including the lie scale would be recommended to examine the respondents’ truthfulness in their self-assessment.

Some of the more interesting findings of the study show the difference between the mental condition and the sleep quality of the participants. The highest rate of sleep disturbances was observed within the group that felt lonely and “couldn’t function”. It would be consistent with an Italian study suggesting that shorter sleeping time was associated with less energy to perform daily activities, and therefore, it could be correlated with a feeling to be unable to function properly [15]. Interestingly, the latter group also differed, with a higher rate of daytime dysfunctions and lower scores of using sleep medication, which can pose a question regarding whether they would not have performed better if they had sought medical help and used some sleep medication. Moreover, lonely participants experienced the greatest troubles with sleep latency and the worst total sleep quality in 2020. Other studies present the same findings of associating loneliness with worse overall sleep quality [74].

People who felt happy significantly differed from the rest in various sleep quality categories. They had the lowest rate of sleep disturbances, which aligns with the studies mentioned above, as negative emotions may cause more sleep disturbances. However, they scored high in sleep latency and daytime dysfunctions in 2020 as well as the usage of sleep medication. As the research suggests, the use of sleep medication may be the reason behind the two previously mentioned higher scores, as the medication may cause persistent daytime somnolence [75].

Another statistically significant difference in our study worth mentioning, which was found in 2020, regarded daytime dysfunctions. The lowest rates in this question were obtained by the students living in rural areas and the highest rates were in cities with more than 500,000 inhabitants. We believe it can be connected with multiple factors. One of them is the difference in the amount of light present during sleeping time. In villages, there is less light pollution from traffic or building lights versus the large agglomerations As light pollution has been proven to cause shallow sleep, therefore, it can increase the sense of sleepiness during the day [76]. Another possible reason behind this discovery may be the difference in noise pollution, as it is more prevalent in urban areas compared to the rural ones [77,78]. Multiple studies conclude similar findings, stating that residents of greater and higher dense population areas are exposed to noise levels that put them at risk of having prominent sleep disturbances [79,80]. Differences in air quality between those areas may also influence sleep disturbances that are more prominent in residents of more polluted urban areas [81]. Additionally, based on the previous study results [82], it is rather unlikely for the reason behind our finding to be associated with different levels of stress, anxiety, or loneliness in these areas.

Additionally, in 2020, we found a difference between greater daytime dysfunctions among physically inactive students compared to moderately active participants. It aligns with the research showing a positive impact on sleep quality of moderate physical exercise [83,84]. Moreover, in 2021, we observed another significant difference, which is the worsening sleep duration associated with the increasing BMI factor, which is consistent with recent studies stating that the short sleep duration is strongly connected with the incidence of obesity [85,86].

### 4.5. Correlation between the Sleep Quality, Physical Activity, and Personal Protective Equipment

The work contributes significantly to the understanding of the correlation between the COVID-19 pandemic and physical activity, personal protective equipment, and sleep quality. These correlations have been explored in many other studies. This work brings additional data that can be used in the future. Branda Yee-Man Yu and others proved that in some conditions, PPE may affect the quality of sleep, and specifically, the deficit of face mask and sanitizers could be the cause of increasing insomnia, sleep initiation, and shortened sleep duration since the local outbreak [87]. The low supply of PPE in local stores and their availability to the public turned out to be a big problem during the pandemic. In addition, poor sleep quality can negatively affect alertness and cognitive quality, with PPE having a similar effect [88]. Otherwise, personal protective equipment affects physical activity. Studies conducted by Epstein et al. have shown that wearing a surgical mask or N95 respirator does not affect performance or physiological processes during aerobic exercise. However, physical activity did not stay at the same level during the pandemic as before the pandemic. The benefit of exercise training has a moderate beneficial effect on sleep quality as proven in a study by Pei-Yu Yang et al. Despite not identifying a specific cause behind this mechanism, the results were evident, and some positive effect on sleep was noted with increasing physical activity [89]. Moreover, maintaining physical activity along with ensuring adequate sleep quality has been proved to have a positive effect on cognitive functions [90].

### 4.6. Research Group Significance

Examining relationships between all the factors mentioned above amongst medical students, in particular, is important as they present a distinct social group that can be later compared to other groups in the future research. Medical students could have been significantly exposed to threats carried by the pandemic due to several reasons. Firstly, they are constantly in a process of learning how to behave responsibly in contact with patients, and yet many of them had been participating in classes at hospitals during the pandemic as well, which could have put their own health at even greater risk [91,92]. Furthermore, medical students as a whole group were more likely to strictly comply with all of the government restrictions as conscientiousness, agreeableness, and neuroticism are some of their personality characteristics [93,94], and these are also associated with a higher rate of compliance to the pandemic restrictions [95]. This could have contributed to the decreased level of physical activity due to a switch to an even more sedentary lifestyle. Moreover, their future professions in medical fields will require of them a certain level of social responsibility which places a high sense of pressure on these students [96]. During the COVID-19 pandemic, all these factors combined could have consequently caused an even higher increased level of stress, anxiety, and depression within this group compared to others affected by the pandemic; however, adverse effects of this period of time were present in different social groups as well.

### 4.7. Limitations of the Study

The presented paper has demonstrated certain changes and effects related to COVID-19, whereas they may be the outcomes of different societal circumstances; therefore, our findings are only of a cross-sectional and informative character. The research group represents merely a small part of society as it consists only of Wroclaw Medical University students. Moreover, due to a few male respondents, the obtained statistics (assigned to male students) might not be the most accurate depiction of their lifestyle, routines, and general condition. As to limitations concerning students’ physical activity, it is crucial to understand that the majority of the study’s respondents were in the first year of faculty, and therefore, they were obligated to participate in Physical Education classes. This might have had an impact on the responses to the students’ exercise habits, which might have been inaccurate and subjective. Another limitation regards the online survey, as students might have misunderstood some of the questions and choose answers that are incompatible with the reality; despite prior instructions given by the researchers who tried to eliminate such situations. Due to the aforementioned, the impact of COVID-19 on the behavioral characteristics evaluated in the study should not be generalized to the adult population. Thus, it would be advised to carry out more research on a broadened group to expand the finding of this paper.

### 4.8. Future Perspectives

Based on our research, it would be beneficial to introduce some countermeasures to deal with poor sleep quality and insufficient physical activity of students. Educational programs could be organized to inform especially women about healthier sleeping hygiene, as they performed significantly worse than men, which would encourage this group, in particular, to be more mindful when it comes to their nocturnal habits. Implementing some practical courses about more appealing ways of intensive physical exercise for female students would be recommended as well. It would be also convenient to carry out another research examining the reasons behind their weakened engagement in a vigorous type of physical activity, so that the causes could be eliminated more effectively. There are several other potential approaches that could be taken to address these issues, which include implementing more structured physical education classes in universities to encourage regular exercise and promote healthy habits; offering resources and support for students to manage with stress and anxiety, which can often contribute to poor sleep quality; and collaborating with local fitness centers or recreational facilities to offer discounted memberships or classes specifically for students in order to make physical activity more attainable. We believe that young adults in general would benefit from learning more about healthy exercising and sleeping routines as these have been disturbed during the pandemic.

## 5. Conclusions

The presented results of the survey allowed drawing several conclusions based on the respondents’ answers. Comparing the period of October 2020 and March 2021, the number of people with BMI level other than normal remains constant, but this group was made up of people with too high BMI in 2021 than it was in 2020. This information is worth attention and dedication in future research. Mental health has changed for the worse, which could be caused by the pandemic situation in the country and the world. There is a prominent level of use of personal protective equipment among medical students, which may be related to the field of study that probably contributes to a greater level of awareness; however, it is a field of research that can be expanded in further work. Comparing physical activity in both periods of the study, as well as between genders, the results indicated an advantage of the average MET score obtained by men as well as an increase in walking activity among students of the medical university during the pandemic. However, the period of the pandemic increased the percentage of physically inactive students and decreased the number of people characterized by the level of physical activity recommended for enhancing health. The effects of this phenomenon were likely to affect the physical and mental health of those surveyed. The period of the pandemic also significantly affected the quality and length of sleep. Women were particularly affected, but this result may be due to the gender disproportion among the respondents (in favor of women). Some dependencies of some emotional states on the subjective assessment of sleep quality have also been shown, which is an interesting topic for further consideration. Sleep hygiene itself is a topic that could be addressed in preventive health care on a larger scale to reduce the percentage of people whose sleep quality is poor; however, sleep quality is not the factor defining the impact of the pandemic on well-being. The work showed us numerous dependencies that may be the direction of further research to increase awareness of the impact of the pandemic on various health aspects. Some of the results may be further investigated and analyzed in the future.

## Figures and Tables

**Table 1:** Characteristics of study participants.

Variables	October 2020, *n* = 696 (%)	March 2021, *n* = 652 (%)
*Age*	20.2 ± 1.6	20.4 ± 1.8
*Gender*		
Male	169 (24.3)	130 (19.9)
Female	527 (75.7)	522 (80.1)
*Place of residence*		
Rural area	186 (26.7)	191 (29.3)
City <20,000 *	72 (10.3)	63 (9.7)
City 20,000–50,000 *	80 (11.5)	73 (11.2)
City 50,000–100,000 *	73 (10.5)	72 (11.0)
City 100,000–500,000 *	79 (11.4)	54 (8.3)
City 500,000+ *	206 (29.6)	199 (30.5)
*BMI*		
Mean	21.5 ± 3.1	21.9 ± 3.4
Underweight (<18.5)	81 (12.0)	68 (10.0)
Normal (18.5–24.9)	543 (78.0)	487 (75.0)
Overweight (>25)	72 (10.0)	97 (15.0)
*Faculty*		
Medical laboratory	41 (5.9)	29 (4.4)
Dietetics	31 (4.5)	33 (5.1)
Pharmacy	128 (18.4)	112 (17.2)
Physiotherapy	78 (11.2)	69 (10.6)
Medicine	329 (47.3)	184 (28.2)
Dentistry	10 (1.4)	27 (4.1)
Nursing	22 (3.2)	128 (19.6)
Midwifery	17 (2.4)	25 (3.8)
Paramedic	28 (4.0)	24 (3.7)
Public health	12 (1.7)	21 (3.2)
*Impact of the pandemic on well-being*		
Positive	18 (2.6)	19 (2.9)
Negative	51 (7.3)	47 (7.2)
None	242 (34.8)	266 (40.8)
Hard to decide	385 (55.3)	320 (49.1)
*Mental condition*		
I feel the same	106 (15.2)	150 (23.0)
I feel lonely	53 (7.6)	60 (9.2)
I feel anxious	85 (12.2)	32 (4.9)
I am more stressed	0 (0.0)	79 (12.1)
I cannot function	52 (7.5)	42 (6.4)
I am overwhelmed	351 (50.4)	242 (37.1)
I feel happy	49 (7.0)	47 (7.2)

**Note**: *n* is the number of observations; * Number of inhabitants.

**Table 2:** Personal Protective Equipment use between the study periods.

PPE Form	October 2020, *n* = 696 (%)	March 2021, *n* = 652 (%)
Washing hands	682 (98.0)	644 (98.8)
Using hand sanitizers	629 (90.4)	571 (87.6)
Using face mask outside the home	690 (99.1)	637 (97.7)
Using gloves outside the home	123 (17.7)	85 (13.0)
Using goggles	13 (1.9)	4 (0.6)
Using gloves inside the home	1 (0.1)	4 (0.6)
Using face mask inside the home	4 (0.6)	7 (1.1)
I do not use any	0 (0.0)	0 (0.0)

**Table 3:** Comparison of physical activity levels between the study periods.

Variables	October 2020, *n* = 696	March 2021, *n* = 652	*p*-Value
*Physical activity MET m*/*w*^1^			
Walking MET m/w	840.8 ^2^ (627 ^3^)	1181.3 (792)	*p* < 0.0001
Moderate MET m/w	139.1 (120)	138.0 (100)	*p* = 0.053
Vigorous MET m/w	635.8 (240)	546.7 (160)	*p* = 0.023
Total MET m/w	1615.7 (1332)	1865.9 (1386)	*p* = 0.0051
*Physical activity level*			
Inactive	541 ^4^ (77.7 ^5^)	526 (80.7)	
Minimally active	74 (10.6)	63 (9.7)	
HEPA	81 (11.6)	63 (9.7)	

**Note**: ^1^—minutes a week; ^2^—mean; ^3^—median; ^4^—*n*; ^5^—percentage.

**Table 4:** Comparison of physical activity levels between genders and the study periods.

Variables	October 2020, *n* = 696	March 2021, *n* = 652	*p*-Value ^4^
*Walking MET m/w* ^1^			
Female	835.0 ^2^ (594 ^3^)	1207.1 (792)	*p* < 0.0001
Male	819.2 (660)	1077.3 (693)	*p* = 0.019
*p*-Value ^5^	*p* = 0.22	*p* = 0.25	
*Moderate MET m/w*			
Female	135.8 (120)	129.2 (100)	*p* = 0.023
Male	149.6 (120)	173.3 (120)	*p* = 0.33
*p*-Value	*p* = 0.47	*p* = 0.038	
*Vigorous MET m*/*w*			
Female	635.8 (240)	490.3 (0)	*p* = 0.025
Male	837.3 (360)	773.3 (400)	*p* = 0.039
*p*-Value	*p* = 0.008	*p* = 0.0006	
*Total MET m/w*			
Female	1542.5 (1258)	1826.6 (1383)	*p* = 0.0077
Male	1845.8 (1440)	2040.7 (1635)	*p* = 0.15
*p*-Value	*p* = 0.009	*p* = 0.025	

**Note**: ^1^—minutes a week; ^2^—mean; ^3^—median; ^4^—comparison between the study periods; ^5^—comparison between the genders.

**Table 5:** Comparison of sleeping quality between the study periods.

Variables	October 2020, *n* = 696	March 2021, *n* = 652	*p*-Value
*Sleep Quality Score*			
Sleep duration	0.37 ± 0.7	0.44 ± 0.74	*p* = 0.022
Sleep disturbances	1.16 ± 0.7	1.21 ± 0.73	*p* = 0.054
Sleep latency	1.41 ± 0.99	1.46 ± 1.00	*p* = 0.20
Daytime dysfunctions	1.53 ± 1.01	1.45 ± 1.05	*p* = 0.085
Habitual sleep efficiency	0.34 ± 0.68	0.36 ± 0.72	*p* = 0.42
Subjective sleep quality	0.92 ± 0.65	0.95 ± 0.62	*p* = 0.22
Use of sleep medication	0.17 ± 0.57	0.16 ± 0.62	*p* = 0.097
Total score	5.90 ± 3.02	6.03 ± 3.27	*p* = 0.33
*Sleep Quality Assessment*			
Good sleep (<5 points)	321 (46.0)	314 (48.0)	
Poor sleep (≥5 points)	375 (54.0)	338 (52.0)	

**Table 6:** Comparison of sleep quality between genders and the study periods.

Variables	October 2020, *n* = 696	March 2021, *n* = 652	*p*-Value ^1^
*Sleep duration*			
Female	0.35	0.43	*p* = 0.029
Male	0.42	0.48	*p* = 0.22
*p*-Value ^2^	*p* = 0.25	*p* = 0.37	
*Sleep disturbances*			
Female	1.18	1.25	*p* = 0.044
Male	1.09	1.09	*p* = 0.43
*p*-Value	*p* = 0.04	*p* = 0.0046	
*Sleep latency*			
Female	1.40	1.44	*p* = 0.28
Male	1.46	1.55	*p* = 0.19
*p*-Value	*p* = 0.27	*p* = 0.14	
*Daytime dysfunctions*			
Female	1.60	1.53	*p* = 0.13
Male	1.30	1.12	*p* = 0.07
*p*-Value	*p* = 0.0009	*p* < 0.0001	
*Habitual sleep efficiency*			
Female	0.37	0.36	*p* = 0.28
Male	0.25	0.36	*p* = 0.07
*p*-Value	*p* = 0.0069	*p* = 0.49	
*Subjective sleep quality*			
Female	0.90	0.94	*p* = 0.21
Male	0.96	0.96	*p* = 0.43
*p*-Value	*p* = 0.33	*p* = 0.47	
*Use of sleep medication*			
Female	0.17	0.17	*p* = 0.20
Male	0.16	0.13	*p* = 0.11
*p*-Value	*p* = 0.50	*p* = 0.38	
*Total score*			
Female	5.98	6.11	*p* = 0.35
Male	5.64	5.70	*p* = 0.46
*p*-Value	*p* = 0.071	*p* = 0.079	

**Note**: ^1^—comparison between the study periods; ^2^—comparison between the genders.

**Table 7:** Comparison of the impact of the pandemic on well-being between the study periods.

Impact of the Pandemic on Well-Being	October 2020, *n* = 696	March 2021, *n* = 652
*Sleep duration*		
Positive	0.47	0.37
Negative	0.20	0.36
None	0.35	0.44
Hard to decide	0.40	0.45
*p*-Value	*p* = 0.18	*p* = 0.85
*Sleep disturbances*		
Positive	1.00	1.32
Negative	0.96	1.13
None	1.12	1.10
Hard to decide	1.22	1.33
*p*-Value	*p* = 0.048	*p* = 0.0026
*Sleep latency*		
Positive	1.76	1.26
Negative	1.12	1.49
None	1.39	1.41
Hard to decide	1.45	1.51
*p*-Value	*p* = 0.056	*p* = 0.46
*Daytime dysfunctions*		
Positive	1.12	1.47
Negative	0.98	0.94
None	1.49	1.32
Hard to decide	1.64	1.63
*p*-Value	*p* < 0.0001	*p* < 0.0001
*Habitual sleep efficiency*		
Positive	0.12	0.47
Negative	0.25	0.34
None	0.31	0.33
Hard to decide	0.38	0.38
*p*-Value	*p* = 0.17	*p* = 0.79
*Subjective sleep quality*		
Positive	0.71	0.79
Negative	0.65	0.83
None	0.95	0.91
Hard to decide	0.94	1.00
*p*-Value	*p* = 0.0039	*p* = 0.14
*Use of sleep medication*		
Positive	0.41	0.32
Negative	0.12	0.11
None	0.17	0.10
Hard to decide	0.15	0.22
*p*-Value	*p* = 0.45	*p* = 0.03
*Total score*		
Positive	5.59	6.00
Negative	4.27	5.19
None	5.78	5.62
Hard to decide	6.20	6.50
*p*-Value	*p* = 0.00012	*p* = 0.007

**Table 8:** Comparison of mental condition between the study periods.

Mental Condition	October 2020, *n* = 696	March 2021, *n* = 652
*Sleep duration*		
I feel the same	0.25	0.39
I feel lonely	0.49	0.52
I feel anxious	0.40	0.25
I am more stressed	0.00	0.62
I cannot function	0.42	0.55
I am overwhelmed	0.37	0.40
I feel happy	0.37	0.43
*p*-Value	*p* = 0.28	*p* = 0.13
*Sleep disturbances*		
I feel the same	1.05	1.02
I feel lonely	1.17	1.40
I feel anxious	1.28	1.31
I am more stressed	0.00	1.23
I cannot function	1.08	1.52
I am overwhelmed	1.18	1.24
I feel happy	1.16	1.13
*p*-Value	*p* = 0.17	*p* = 0.00076
*Sleep latency*		
I feel the same	1.33	1.30
I feel lonely	1.92	1.58
I feel anxious	1.53	1.66
I am more stressed	0.00	1.47
I cannot function	1.31	1.71
I am overwhelmed	1.32	1.46
I feel happy	1.61	1.45
*p*-Value	*p* = 0.00073	*p* = 0.29
*Daytime dysfunctions*		
I feel the same	1.10	1.04
I feel lonely	1.62	1.55
I feel anxious	1.47	1.22
I am more stressed	0.00	1.61
I cannot function	1.67	1.86
I am overwhelmed	1.60	1.57
I feel happy	1.78	1.47
*p*-Value	*p* = 0.00011	*p* < 0.0001
*Habitual sleep efficiency*		
I feel the same	0.29	0.25
I feel lonely	0.45	0.23
I feel anxious	0.31	0.31
I am more stressed	0.00	0.43
I cannot function	0.29	0.48
I am overwhelmed	0.37	0.40
I feel happy	0.27	0.49
*p*-Value	*p* = 0.42	*p* = 0.29
*Subjective sleep quality*		
I feel the same	0.84	0.79
I feel lonely	0.98	0.98
I feel anxious	0.91	0.78
I am more stressed	0.00	1.22
I cannot function	0.96	1.14
I am overwhelmed	0.94	0.92
I feel happy	0.84	1.06
*p*-Value	*p* = 0.51	*p* = 0.00028
*Use of sleep medication*		
I feel the same	0.16	0.13
I feel lonely	0.19	0.18
I feel anxious	0.25	0.13
I am more stressed	0.00	0.16
I cannot function	0.04	0.19
I am overwhelmed	0.13	0.17
I feel happy	0.39	0.19
*p*-Value	*p* = 0.029	*p* = 0.99
*Total score*		
I feel the same	5.03	4.37
I feel lonely	6.83	5.73
I feel anxious	6.14	4.84
I am more stressed	0.00	6.04
I cannot function	5.77	6.64
I am overwhelmed	5.91	5.48
I feel happy	6.41	5.55
*p*-Value	*p* = 0.00097	*p* < 0.0001

**Table 9:** Comparison of sleep quality and place of residency between study periods.

Variables	October 2020, *n* = 696	March 2021, *n* = 652
*Sleep duration*		
Rural area	0.40	0.51
City <20,000 *	0.26	0.24
City 20,000–50,000 *	0.34	0.47
City 50,000–100,000 *	0.47	0.46
City 100,000–500,000 *	0.35	0.41
City 500,000+ *	0.36	0.42
*p*-Value	*p* = 0.58	*p* = 0.31
*Sleep disturbances*		
Rural area	1.08	1.14
City <20,000 *	1.17	1.13
City 20,000—50,000 *	1.23	1.15
City 50,000—100,000 *	1.32	1.35
City 100,000—500,000 *	1.18	1.11
City 500,000+ *	1.15	1.33
*p*-Value	*p* = 0.27	*p* = 0.055
*Sleep latency*		
Rural area	1.32	1.35
City <20,000 *	1.46	1.41
City 20,000–50,000 *	1.51	1.42
City 50,000–100,000 *	1.47	1.54
City 100,000–500,000 *	1.43	1.61
City 500,000+ *	1.42	1.52
*p*-Value	*p* = 0.73	*p* = 0.48
*Daytime dysfunctions*		
Rural area	1.34	1.39
City <20,000 *	1.51	1.13
City 20,000–50,000 *	1.53	1.41
City 50,000–100,000 *	1.60	1.68
City 100,000–500,000 *	1.58	1.56
City 500,000+ *	1.66	1.50
*p*-Value	*p* = 0.043	*p* = 0.044
*Habitual sleep efficiency*		
Rural area	0.35	0.35
City <20,000 *	0.32	0.38
City 20,000–50,000 *	0.38	0.41
City 50,000–100,000 *	0.33	0.42
City 100,000–500,000 *	0.28	0.28
City 500,000+ *	0.35	0.35
*p*-Value	*p* = 0.64	*p* = 0.68
*Subjective sleep quality*		
Rural area	0.80	0.91
City <20,000 *	0.93	0.83
City 20,000–50,000 *	1.01	0.96
City 50,000–100,000 *	1.03	1.04
City 100,000–500,000 *	0.87	1.06
City 500,000+ *	0.96	0.95
*p*-Value	*p* = 0.06	*p* = 0.49
*Use of sleep medication*		
Rural area	0.10	0.06
City <20,000 *	0.19	0.08
City 20,000–50,000 *	0.21	0.21
City 50,000–100,000 *	0.03	0.22
City 100,000–500,000 *	0.32	0.17
City 500,000+ *	0.19	0.25
*p*-Value	*p* = 0.016	*p* = 0.048
*Total score*		
Rural area	5.39	5.71
City <20,000 *	5.85	5.19
City 20,000–50,000 *	6.20	6.03
City 50,000–100,000 *	6.23	6.71
City 100,000–500,000 *	6.01	6.19
City 500,000+ *	6.09	6.33
*p*-Value	*p* = 0.11	*p* = 0.07

* Number of inhabitants.

## Data Availability

The datasets used and/or analyzed during the current study are available from the corresponding author upon reasonable request.

## References

[1] Hui D.S., Azhar E.I., Madani T.A., Ntoumi F., Kock R., Dar O., Ippolito G., Mchugh T.D., Memish Z.A., Drosten C. (2020). The Continuing 2019-NCoV Epidemic Threat of Novel Coronaviruses to Global Health—The Latest 2019 Novel Coronavirus Outbreak in Wuhan, China. Int. J. Infect. Dis..

[2] Lu H., Stratton C.W., Tang Y.W. (2020). Outbreak of Pneumonia of Unknown Etiology in Wuhan, China: The Mystery and the Miracle. J. Med. Virol..

[3] WHO WHO Director-General’s Opening Remarks at the Media Briefing on COVID-19—11 March 2020. https://www.who.int/director-general/speeches/detail/who-director-general-s-opening-remarks-at-the-media-briefing-on-covid-19---11-march-2020.

[4] Rozporządzenie Ministra Zdrowia z Dnia 20 Marca 2020 r. w Sprawie Ogłoszenia Na Obszarze Rzeczypospolitej Polskiej Stanu Epidemii. https://isap.sejm.gov.pl/isap.nsf/DocDetails.xsp?id=WDU20200000491.

[5] Buheji M., Jahrami H., Sabah Dhahi A. (2020). Minimising Stress Exposure during Pandemics Similar to COVID-19. Int. J. Psychol. Behav. Sci..

[6] Brooks S.K., Webster R.K., Smith L.E., Woodland L., Wessely S., Greenberg N., Rubin G.J. (2020). The Psychological Impact of Quarantine and How to Reduce It: Rapid Review of the Evidence. Lancet.

[7] Javed B., Sarwer A., Soto E.B., Mashwani Z.u.R. (2020). The Coronavirus (COVID-19) Pandemic’s Impact on Mental Health. Int. J. Health Plan. Manag..

[8] Coronavirus. https://www.who.int/health-topics/coronavirus#tab=tab_1.

[9] Advice for the Public. https://www.who.int/emergencies/diseases/novel-coronavirus-2019/advice-for-public.

[10] Królikowski K., Fiedur M. (2022). Postępowanie Studentów w Zakresie Stosowania Środków Ochrony Osobistej w Okresie Pandemii COVID-19. Pol. J. Sustain. Dev..

[11] #zostań w Domu, Bądź Bezpieczny—Komenda Powiatowa Państwowej Straży Pożarnej w Turku—Portal Gov.Pl. https://www.gov.pl/web/kppsp-turek/zostan-w-domu-badz-bezpieczny?fbclid=IwAR1U1ZHugKpA_IpAUGsrft7lG3UyzlcT3EpmYwmnVt8vrMW8zCB4ExXvKZo.

[12] Silva R.R., Rufino C.R., Galvão L.L., Vancini R.L., Santos D.A.T., de Lira C.A.B., Andrade M.S., Nikolaidis P.T., Okuno M.F.P., dos Santos R.G. (2022). Motivation for Brazilian Older Adult Women to Join a Community Physical Activity Program before COVID-19 Pandemic. Int. J. Sport Stud. Health.

[13] Kaur H., Singh T., Arya Y.K., Mittal S. (2020). Physical Fitness and Exercise During the COVID-19 Pandemic: A Qualitative Enquiry. Front. Psychol..

[14] Castañeda-Babarro A., Coca A., Arbillaga-Etxarri A., Gutiérrez-Santamaría B. (2020). Physical Activity Change during COVID-19 Confinement. Int. J. Environ. Res. Public Health.

[15] Luciano F., Cenacchi V., Vegro V., Pavei G. (2021). COVID-19 Lockdown: Physical Activity, Sedentary Behaviour and Sleep in Italian Medicine Students. Eur. J. Sport Sci..

[16] Özden G., Parlar Kiliç S. (2021). The Effect of Social Isolation during COVID-19 Pandemic on Nutrition and Exercise Behaviors of Nursing Students. Ecol. Food Nutr..

[17] Hegberg N.J., Tone E.B. (2015). Physical Activity and Stress Resilience: Considering Those at-Risk for Developing Mental Health Problems. Ment. Health Phys. Act..

[18] Gerber M., Jonsdottir I.H., Lindwall M., Ahlborg G. (2014). Physical Activity in Employees with Differing Occupational Stress and Mental Health Profiles: A Latent Profile Analysis. Psychol. Sport Exerc..

[19] Sousa C.V., Sales M., de Moraes J.F.V.N., de Oliveira Rocha P., dos Santos R., Petriz B. (2014). Sedentary Life Style Is Associated with an Elevated Perceived Stress. J. Exerc. Physiol. Online.

[20] Lee E., Kim Y. (2019). Effect of University Students’ Sedentary Behavior on Stress, Anxiety, and Depression. Perspect. Psychiatr. Care.

[21] Gupta R., Grover S., Basu A., Krishnan V., Tripathi A., Subramanyam A., Nischal A., Hussain A., Mehra A., Ambekar A. (2020). Changes in Sleep Pattern and Sleep Quality during COVID-19 Lockdown. Indian J. Psychiatry.

[22] Fila-Witecka K., Senczyszyn A., Kołodziejczyk A., Ciułkowicz M., Maciaszek J., Misiak B., Szcześniak D., Rymaszewska J. (2021). Lifestyle Changes among Polish University Students during the COVID-19 Pandemic. Int. J. Environ. Res. Public Health.

[23] de Sousa Martins e Silva E., Ono B.H.V.S., Souza J.C. (2020). Sleep and Immunity in Times of COVID-19. Rev. Assoc. Med. Bras..

[24] Irwin M.R. (2015). Why Sleep Is Important for Health: A Psychoneuroimmunology Perspective. Annu. Rev. Psychol..

[25] Medic G., Wille M., Hemels M.E.H. (2017). Short- and Long-Term Health Consequences of Sleep Disruption. Nat. Sci. Sleep.

[26] Azad M.C., Fraser K., Rumana N., Abdullah A.F., Shahana N., Hanly P.J., Turin T.C. (2015). Sleep Disturbances among Medical Students: A Global Perspective. J. Clin. Sleep Med..

[27] Brick C.A., Seely D.L., Palermo T.M. (2010). Association Between Sleep Hygiene and Sleep Quality in Medical Students. Behav. Sleep Med..

[28] Pagnin D., de Queiroz V., Carvalho Y.T.M.S., Dutra A.S.S., Amaral M.B., Queiroz T.T. (2014). The Relation Between Burnout and Sleep Disorders in Medical Students. Acad. Psychiatry.

[29] Kliszcz J., Nowicka-Sauer K., Trzeciak B., Sadowska A. (2004). The Level of Anxiety, Depression and Aggression in Nurses and Their Life and Job Satisfaction. Med. Pr..

[30] Grace M.K., VanHeuvelen J.S. (2019). Occupational Variation in Burnout among Medical Staff: Evidence for the Stress of Higher Status. Soc. Sci. Med..

[31] (2021). Prolonged Stress, Anxiety, and Depression in Medical Staff during the COVID-19 Crisis. Psychosociol. Issues Hum. Resour. Manag..

[32] Darr K. (1997). The Social Responsibility of Hospitals. Hosp. Top..

[33] Dharamsi S., Ho A., Spadafora S.M., Woollard R. (2011). The Physician as Health Advocate: Translating the Quest for Social Responsibility into Medical Education and Practice. Acad. Med..

[34] Francis C.K. (2001). The Medical Ethos and Social Responsibility in Clinical Medicine. J. Natl. Med. Assoc..

[35] Biernat E., Stupnicki R., Gajewski A. (2007). International Physical Activity Questionnaire (IPAQ)—Polish Version. Wych. Fiz. Sport.

[36] IPAQ Scoring Protocol—International Physical Activity Questionnaire. https://sites.google.com/site/theipaq/scoring-protocol.

[37] Buysse D.J., Reynolds C.F., Monk T.H., Berman S.R., Kupfer D.J. (1989). The Pittsburgh Sleep Quality Index: A New Instrument for Psychiatric Practice and Research. Psychiatry Res..

[38] Ludnościowa R.R. (2020). Sytuacja Demograficzna Polski. Raport 2019–2020. https://bip.stat.gov.pl/download/gfx/bip/pl/defaultstronaopisowa/805/1/1/rrl_sytuacja_demograficzna_polski_-_raport_2019-2020_final.pdf.

[39] Birmingham W.C., Wadsworth L.L., Lassetter J.H., Graff T.C., Lauren E., Hung M. (2021). COVID-19 Lockdown: Impact on College Students’ Lives. J. Am. Coll. Health.

[40] Sidor A., Rzymski P. (2020). Dietary Choices and Habits during COVID-19 Lockdown: Experience from Poland. Nutrients.

[41] Sumalla-Cano S., Forbes-Hernández T., Aparicio-Obregón S., Crespo J., Eléxpuru-Zabaleta M., Gracia-Villar M., Giampieri F., Elío I. (2022). Changes in the Lifestyle of the Spanish University Population during Confinement for COVID-19. Int. J. Environ. Res. Public Health.

[42] Cawley J., Burkhauser R.v., Hall M. (2006). Beyond BMI: The Value of More Accurate Measures of Fatness and Obesity in Social Science Research. J. Health Econ..

[43] Ferrante G., Rossini P.G., Rousset S., Ostacoli L., Piccinelli C., Carletto S., Giordano L. (2022). The Emotional Side of Post-Traumatic Stress Reaction during COVID-19 Pandemic: An Italian Survey. BMC Public Health.

[44] Aslan H., Pekince H. (2021). Nursing Students’ Views on the COVID-19 Pandemic and Their Percieved Stress Levels. Perspect. Psychiatr. Care.

[45] Shah S.M.A., Mohammad D., Qureshi M.F.H., Abbas M.Z., Aleem S. (2021). Prevalence, Psychological Responses and Associated Correlates of Depression, Anxiety and Stress in a Global Population, During the Coronavirus Disease (COVID-19) Pandemic. Community Ment. Health J..

[46] Kiedy Nosić Maseczkę?|Pacjent. https://pacjent.gov.pl/aktualnosc/kiedy-nosic-maseczke.

[47] Oliveira R., Plácido M., Pereira L., Machado S., Parreira E., Gil-Gouveia R. (2022). Headaches and the Use of Personal Protective Equipment in the General Population during the COVID-19 Pandemic. Cephalalgia.

[48] Pittet D. (2000). Improving Compliance with Hand Hygiene in Hospitals. Infect. Control Hosp. Epidemiol..

[49] Erasmus V., Brouwer W., van Beeck E.F., Oenema A., Daha T.J., Richardus J.H., Vos M.C., Brug J. (2009). A Qualitative Exploration of Reasons for Poor Hand Hygiene Among Hospital Workers Lack of Positive Role Models and of Convincing Evidence That Hand Hygiene Prevents Cross-Infection. Infect. Control Hosp. Epidemiol..

[50] Xu H., Gan Y., Zheng D., Wu B., Zhu X., Xu C., Liu C., Tao Z., Hu Y., Chen M. (2020). Relationship Between COVID-19 Infection and Risk Perception, Knowledge, Attitude, and Four Nonpharmaceutical Interventions During the Late Period of the COVID-19 Epidemic in China: Online Cross-Sectional Survey of 8158 Adults. J. Med. Internet Res..

[51] Doung-ngern P., Suphanchaimat R., Panjangampatthana A., Janekrongtham C., Ruampoom D., Daochaeng N., Eungkanit N., Pisitpayat N., Srisong N., Yasopa O. (2020). Case-Control Study of Use of Personal Protective Measures and Risk for SARS-CoV 2 Infection, Thailand. Emerg. Infect. Dis..

[52] Sekulic M., Stajic D., Jurisic Skevin A., Kocovic A., Zivkovic Zaric R., Djonovic N., Vasiljevic D., Radmanovic B., Spasic M., Janicijevic K. (2022). Lifestyle, Physical Activity, Eating and Hygiene Habits: A Comparative Analysis Before and During the COVID-19 Pandemic in Student Population. Front. Public Health.

[53] Franco I., Bianco A., Bonfiglio C., Sorino P., Mirizzi A., Campanella A., Buongiorno C., Liuzzi R., Osella A.R. (2021). Decreased levels of physical activity: Results from a cross-sectional study in southern Italy during the COVID-19 lockdown. J. Sports Med. Phys. Fit..

[54] Yang Y., Koenigstorfer J. (2020). Determinants of Physical Activity Maintenance during the COVID-19 Pandemic: A Focus on Fitness Apps. Transl. Behav. Med..

[55] Romero-Blanco C., Rodríguez-Almagro J., Onieva-Zafra M.D., Parra-Fernández M.L., Prado-Laguna M.d.C., Hernández-Martínez A. (2020). Physical Activity and Sedentary Lifestyle in University Students: Changes during Confinement Due to the COVID-19 Pandemic. Int. J. Environ. Res. Public Health.

[56] Puccinelli P.J., da Costa T.S., Seffrin A., de Lira C.A.B., Vancini R.L., Nikolaidis P.T., Knechtle B., Rosemann T., Hill L., Andrade M.S. (2021). Reduced Level of Physical Activity during COVID-19 Pandemic Is Associated with Depression and Anxiety Levels: An Internet-Based Survey. BMC Public Health.

[57] Aegerter A.M., Deforth M., Sjøgaard G., Johnston V., Volken T., Luomajoki H., Dratva J., Dressel H., Distler O., Melloh M. (2021). No Evidence for a Decrease in Physical Activity Among Swiss Office Workers During COVID-19: A Longitudinal Study. Front. Psychol..

[58] Olfert M.D., Wattick R.A., Saurborn E.G., Hagedorn R.L. (2022). Impact of COVID-19 on College Student Diet Quality and Physical. Nutr. Health.

[59] Ammar A., Brach M., Trabelsi K., Chtourou H., Boukhris O., Masmoudi L., Bouaziz B., Bentlage E., How D., Ahmed M. (2020). Effects of COVID-19 Home Confinement on Eating Behaviour and Physical Activity: Results of the ECLB-COVID19 International Online Survey. Nutrients.

[60] López-Valenciano A., Suárez-Iglesias D., Sanchez-Lastra M.A., Ayán C. (2021). Impact of COVID-19 Pandemic on University Students’ Physical Activity Levels: An Early Systematic Review. Front. Psychol..

[61] Wattanapisit A., Fungthongcharoen K., Saengow U., Vijitpongjinda S. (2016). Physical Activity among Medical Students in Southern Thailand: A Mixed Methods Study. BMJ Open.

[62] Frank E., Tong E., Lobelo F., Carrera J., Duperly J. (2008). Physical Activity Levels and Counseling Practices of U.S. Medical Students. Med. Sci. Sports Exerc..

[63] Blake H., Stanulewicz N., McGill F. (2017). Predictors of Physical Activity and Barriers to Exercise in Nursing and Medical Students. J. Adv. Nurs.

[64] da Silva B.B.L., de Melo M.C.F., Studart-Pereira L.M. (2022). Adolescents’ Sleep Quality during the COVID-19 Pandemic. Sleep Sci..

[65] Madrid-Valero J.J., Martínez-Selva J.M., Ribeiro do Couto B., Sánchez-Romera J.F., Ordoñana J.R. (2017). Age and Gender Effects on the Prevalence of Poor Sleep Quality in the Adult Population. Gac. Sanit..

[66] Li L., Sheehan C.M., Thompson M.S. (2019). Measurement Invariance and Sleep Quality Differences Between Men and Women in the Pittsburgh Sleep Quality Index. J. Clin. Sleep Med..

[67] Eleftheriou A., Rokou A., Arvaniti A., Nena E., Steiropoulos P. (2021). Sleep Quality and Mental Health of Medical Students in Greece During the COVID-19 Pandemic. Front. Public Health.

[68] Tahir M.J., Malik N.I., Ullah I., Khan H.R., Perveen S., Ramalho R., Siddiqi A.R., Waheed S., Shalaby M.M.M., de Berardis D. (2021). Internet Addiction and Sleep Quality among Medical Students during the COVID-19 Pandemic: A Multinational Cross-Sectional Survey. PLoS ONE.

[69] Trabelsi K., Ammar A., Masmoudi L., Boukhris O., Chtourou H., Bouaziz B., Brach M., Bentlage E., How D., Ahmed M. (2021). Sleep Quality and Physical Activity as Predictors of Mental Wellbeing Variance in Older Adults during COVID-19 Lockdown: ECLB COVID-19 International Online Survey. Int. J. Environ. Res. Public Health.

[70] Bloom H.G., Ahmed I., Alessi C.A., Ancoli-Israel S., Buysse D.J., Kryger M.H., Phillips B.A., Thorpy M.J., Vitiello M.v., Zee P.C. (2009). Evidence-Based Recommendations for the Assessment and Management of Sleep Disorders in Older Persons. J. Am. Geriatr. Soc..

[71] Dijk D.-J., Groeger J.A., Stanley N., Deacon S. (2010). Age-Related Reduction in Daytime Sleep Propensity and Nocturnal Slow Wave Sleep. Sleep.

[72] Sher L. (2020). COVID-19, Anxiety, Sleep Disturbances and Suicide. Sleep Med..

[73] Walker G., McCabe T. (2021). Psychological Defence Mechanisms during the COVID-19 Pandemic: A Case Series. Eur. J. Psychiatry.

[74] Matthews T., Danese A., Gregory A.M., Caspi A., Moffitt T.E., Arseneault L. (2017). Sleeping with One Eye Open: Loneliness and Sleep Quality in Young Adults. Psychol. Med..

[75] Pagel J.F. (2005). Medications and Their Effects on Sleep. Prim. Care Clin. Off. Pract..

[76] Cho J.R., Joo E.Y., Koo D.L., Hong S.B. (2013). Let There Be No Light: The Effect of Bedside Light on Sleep Quality and Background Electroencephalographic Rhythms. Sleep Med..

[77] Firdaus G., Ahmad A. (2010). Noise Pollution and Human Health: A Case Study of Municipal Corporation of Delhi. Indoor Built Environ..

[78] Olayinka Oyedepo S. (2012). Noise Pollution in Urban Areas: The Neglected Dimensions. Environ. Res. J..

[79] Cho D.-H., Lee S.J., Jae S.Y., Kim W.J., Ha S.J., Gwon J.G., Choi J., Kim D.W., Kim J.Y. (2021). Physical Activity and the Risk of COVID-19 Infection and Mortality: A Nationwide Population-Based Case-Control Study. J. Clin. Med..

[80] Halperin D. (2014). Environmental Noise and Sleep Disturbances: A Threat to Health?. Sleep Sci..

[81] Xu X., Lian Z. (2022). Objective Sleep Assessments for Healthy People in Environmental Research: A Literature Review. Indoor Air.

[82] Kosendiak A.A., Adamczak B., Bania J., Kontek S. (2022). Stress Levels, Attitude toward Vaccination and Personal Protective Equipment of Students at Wroclaw Medical University during the COVID-19 Pandemic. Int. J. Environ. Res. Public Health.

[83] Reid K.J., Baron K.G., Lu B., Naylor E., Wolfe L., Zee P.C. (2010). Aerobic Exercise Improves Self-Reported Sleep and Quality of Life in Older Adults with Insomnia. Sleep Med..

[84] Wang F., Boros S. (2019). The Effect of Physical Activity on Sleep Quality: A Systematic Review. Eur. J. Physiother..

[85] Wu Y., Zhai L., Zhang D. (2014). Sleep Duration and Obesity among Adults: A Meta-Analysis of Prospective Studies. Sleep Med..

[86] Cappuccio F.P., Taggart F.M., Kandala N.B., Currie A., Peile E., Stranges S., Miller M.A. (2008). Meta-Analysis of Short Sleep Duration and Obesity in Children and Adults. Sleep.

[87] Yu B.Y.-M., Yeung W.-F., Lam J.C.-S., Yuen S.C.-S., Lam S.C., Chung V.C.-H., Chung K.-F., Lee P.H., Ho F.Y.-Y., Ho J.Y.-S. (2020). Prevalence of Sleep Disturbances during COVID-19 Outbreak in an Urban Chinese Population: A Cross-Sectional Study. Sleep Med..

[88] Wells H.J., Raithatha M., Elhag S., Turner J., Osuri P., Kannan S. (2022). Impact of Full Personal Protective Equipment on Alertness of Healthcare Workers: A Prospective Study. BMJ. Open Qual..

[89] Yang P.-Y., Ho K.-H., Chen H.-C., Chien M.-Y. (2012). Exercise Training Improves Sleep Quality in Middle-Aged and Older Adults with Sleep Problems: A Systematic Review. J. Physiother..

[90] Sewell K.R., Erickson K.I., Rainey-Smith S.R., Peiffer J.J., Sohrabi H.R., Brown B.M. (2021). Relationships between Physical Activity, Sleep and Cognitive Function: A Narrative Review. Neurosci. Biobehav. Rev..

[91] Taghrir M.H., Borazjani R., Shiraly R. (2020). COVID-19 and Iranian Medical Students; A Survey on Their Related-Knowledge, Preventive Behaviors and Risk Perception. Arch. Iran. Med..

[92] Alsoghair M., Almazyad M., Alburaykan T., Alsultan A., Alnughaymishi A., Almazyad S., Alharbi M., Alkassas W., Almadud A., Alsuhaibani M. (2021). Medical Students and COVID-19: Knowledge, Preventive Behaviors, and Risk Perception. Int. J. Environ. Res. Public Health.

[93] Lievens F., Coetsier P., de Fruyt F., de Maeseneer J. (2002). Medical Students’ Personality Characteristics and Academic Performance: A Five-Factor Model Perspective. Med. Educ..

[94] Ahmadi J., Pridmore S., Fallahzadeh M. (2004). Neurotic Scores in a Sample of Medical Students. Ger. J. Psychiatry.

[95] Zajenkowski M., Jonason P.K., Leniarska M., Kozakiewicz Z. (2020). Who Complies with the Restrictions to Reduce the Spread of COVID-19?: Personality and Perceptions of the COVID-19 Situation. Personal. Individ. Differ..

[96] Alharbi H., Almalki A., Alabdan F., Haddad B. (2018). Depression among Medical Students in Saudi Medical Colleges: A Cross-Sectional Study. Adv. Med. Educ. Pract..

